# Biomarkers during early healing of extraction procedures: a prospective case series

**DOI:** 10.1007/s00784-025-06534-8

**Published:** 2025-10-06

**Authors:** Alessandra Toti, Ada Kura, Elena Sticchi, Marco Duvina, Vanni Balestri, Franco Amunni, Paolo Tonelli, Lorenzo Di Cesare Mannelli, Carla Ghelardini, Ester Parisi

**Affiliations:** 1 Department of Neuroscience, Psychology, Drug Research and Child Health - NEUROFARBA - Pharmacology and Toxicology Section, University of Florence, Florence, Italy; 2 Department of Experimental and Clinical Medicine, University of Florence, Florence, Italy; 3 Atherothrombotic Center, Department of Experimental and Clinical Medicine, University of Florence, AOU Careggi, Florence, Italy; 4 Department of Oral Surgery, University of Florence School of Dentistry, Florence, Italy; 5 Department of Special Dentistry, Centro Traumatologico Ortopedico, AOU Careggi, Florence, Italy

**Keywords:** Early healing, Tooth extraction, Cytokines, Growth factors

## Abstract

**Objectives:**

This study aims to: (1) evaluate the temporal profiles of VEGF-A, IL-1β, IL-6, IL-10, G-CSF, TNF-α, and β-NGF in surgical wound exudate (WF), comparing them to each patient’s baseline values from gingival crevicular fluid (GCF); (2) describe a non-invasive, reliable method for collecting and processing WF to identify molecular markers involved in wound healing.

**Materials and methods:**

Twenty-four patients underwent surgical tooth extraction. GCF samples were collected preoperatively (T0), and WF samples 24 h (T1) and 7 days (T2) postoperatively. Protein concentrations were measured using multiplex ELISA and expressed as mean ± SEM.

**Results:**

IL-1β significantly increased from 23.58 ± 4.52 (T0) to 83.95 ± 21.39 (T1; *p* < 0.001). TNF-α rose from 1.82 ± 0.48 to 40.88 ± 11.27 (*p* < 0.0001), IL-6 peaked at 214.76 ± 38.21 (from 2.37 ± 0.98; *p* < 0.0001), and VEGF-A increased to 18.50 ± 4.85 (from 3.46 ± 2.19; *p* < 0.001).

**Conclusions:**

The method enabled efficient, non-invasive assessment of protein levels relative to individual baselines, confirming expected biomarker trends in early wound healing.

**Clinical relevance:**

Non-invasive biomarker analysis of wound exudate offers a practical approach to monitor healing and support targeted pain reduction and tissue repair strategies.

## Introduction

Surgical wound healing is a complex phenomenon involving cytokines and growth factors, so that inflammatory and reparative processes coexist [1], initiating cell migration, differentiation and proliferation through a complex reciprocal interaction [2]. Growth factors act as mitogenic and angiogenic signals in the early stages of healing. Once activated, they trigger a series of events through ligand-receptor interactions, including signal transduction, gene transcription, mRNA-directed protein biosynthesis and post-translational protein secretion [3]. In the first phase of wound healing, i.e. clot creation, there is peak secretion of the following growth factors: epidermal growth factor (EGF), platelet-derived growth factor (PDGF), vascular endothelial growth factor (VEGF), transforming growth factor-beta (TGF-β), and basic fibroblast growth factor (bFGF) [4]. These factors attract neutrophils and induce monocyte differentiation.

On the other hand, cytokines, synthesized mainly by neutrophils, monocytes, endothelial cells and fibroblasts, initiate the inflammatory phase, which is concomitant to haemostasis but lasts longer, at least 4–6 days. Cytokines have effects in modulating the inflammatory and immune response. They are produced locally or over short distances and in vivo concentrations range from a few picograms to nanograms per ml. Due to this local action at low concentrations, their serum levels may not reliably reflect local activation [5, 6]. The main cytokines that peak in concentration in the first few hours are tumour necrosis factor-alpha (TNF-α), interleukin-1 beta (IL-1β) and interleukin-6 (IL-6) [7]; they initiate a cascade mechanism by influencing migration, proliferation and differentiation of numerous cells: fibroblasts, epithelial cells, osteoblasts and macrophages, as well as the activation of T- and B-lymphocytes. At the same time, they have been definitively associated with the onset of post-operative pain and hyperalgesia [6, 8] and, in the event of pro- and anti-inflammatory balance dysregulation, they can result in overexpression of pro-inflammatory mediators or negative modulators of regeneration (expression of matrix metalloproteinase MMPs, osteoclastic induction and apoptosis of matrix-producing cells), with worsening of periodontal tissue regeneration [7, 9]. In particular, IL-1β and TNF-α allow the early secretion of pro-inflammatory mediators: specifically, two peaks of cyclooxygenase 2 (COX-2) occur; the first occurs in the first two hours after surgery, with secretion of prostaglandin E2 (PGE2); the second, between 24 and 48 h, with the release of prostaglandin D2 (PGD2), which in turn modulates inflammation by allowing the expression of selective pro-resolving mediators (SPMs) or pro-resolvins, thus reducing the expression of IL-1, IL-6, TNF-α, TGF-β, with a gradual reduction, from the fourth day onwards, of inflammatory events and promotion of the proliferative phase. This is followed by the proliferative phase (from day 4 to day 14, approximately) with the creation of granulation tissue; in this phase, the levels of cytokines are again raised, no longer for pro-inflammatory purposes but for activation and induction towards keratinocytes, fibroblasts and endothelial cells [7]. Transforming growth factor-β (TGF-β) acts as a link between the end of the inflammatory phase and the beginning of the proliferative phase, with a chemotactic effect on epithelial, endothelial and fibroblastic cells and an action that favours osteoclasts and thus bone remodeling [10]. The remodeling phase concludes healing and is characterized by the development of new epithelial tissue, the elimination of granulation tissue and its progressive replacement with connective tissue [11]. Starting at two to three weeks, both IL-1 and TNF-α peak again at early bone remodeling [12]. Together with IL-6 they enable the regulation of both osteoclastic and osteoblastic activity [13]. Finally, among the growth factors, bone morphogenetic protein-4 (BMP-4) appears, which induces osteoblastic differentiation and stimulates mesenchymal stem cell recruitment [14].

Collecting and measuring biochemical markers present at the surgical site can be useful for monitoring the progress of surgical wound healing, assessing the effect of both systemic and local drug administration on cytokine expression, but also for understanding the relationship between mediator release and the occurrence of post-operative pain. In Goto’s review [8] various biomarkers were associated with acute post-operative pain and it was observed that many pro-inflammatory cytokines including ILs, TNF-α, granulocyte colony-stimulating factor (G-CSF), and monocyte chemoattractant protein-1 (MCP-1) are associated with surgical wound pain in caesarean section, in reduction and reconstructive mastoplasty, and in chronic venous leg ulcers. Many studies focus on systemic measurements of molecular mediators without considering site-specific release: instead, serum measurements may not reflect local wound biochemical events and indicate only a global systemic response to surgical injury [15–17].

In assessing the existing inflammatory condition of the periodontal tissues adjacent to an element, gingival crevicular fluid (GCF) could be a valid evaluation parameter.

GCF is a fluid classified as an inflammatory transudate or otherwise an altered tissue transudate in a healthy gingival tissue condition. GCF is released in the gingival sulcus and mirrors the composition of serum because it contains local and migrated inflammatory mediators, serum transudate, subgingival plaque bacteria and cells, both epithelial and leukocytes, and extracellular proteins. However, its composition is variable depending on the health of the periodontal complex. Because of the possibility of non-invasive sampling of GCF, it is normally used as a diagnostic fluid for periodontal diseases, for tissue healing following periodontal surgery or for analyzing the impact of medication on periodontal health. Many methods are available for collecting GCF: paper strips, micropipettes and paper cones. In the last decade, researchers have favoured the use of adsorbent paper cones due to their easy insertion into the sulcus without inducing periodontal bleeding [18, 19].

The aim of the study is (1) to identify the trend profiles of the following proteins in surgical wound exudate (henceforth referred to as Wound Fluid, WF): VEGF-A, IL-1β, IL-6, IL-10, G-CSF, TNF-α, β-NGF, comparing them with the individual baseline values for each patient, obtained from the GCF taken from the gingival sulcus of the tooth element that will undergo avulsion. (2) to describe a method for collecting WF and its processing in order to identify molecular markers expressed during surgical wound healing in a non-invasive and reliable manner.

### Purpose

Using a non-invasive and reliable method for marker assessment during the healing phases could be useful: assessment of biomarker levels in surgical wound exudate can be used to more objectively estimate wound progress and contribute to developing accurate management options to reduce pain and promote healing [8].

### Clinical implications

The methodology of collection, processing and study of biomarkers illustrated here could be useful for future research; evaluation of the physiological or pathological expression of the cytokine cascade in the early stages of healing could help in the development of targeted therapies (e.g. molecularly targeted drugs) [20].

## Materials and methods

This pilot, observational, prospective, single-center study was conducted following the Good Clinical Practice Guidelines (GCPs) and following the STROBE guidelines (https://www.strobe-statement.org/) for observational studies. The study was approved by the Careggi Ethics Committee (21368_bio) and followed the recommendations of the Declaration of Helsinki for studies in human subjects (2013).

### Study population

The sample consists of 24 subjects consecutively enrolled at the Careggi Odontostomatology Clinic and Special Dentistry. All subjects required a tooth extraction that could be defined as ‘surgical’, i.e., involving flap incision and elevation, osteotomy and, when necessary, odontotomy.

#### Inclusion criteria

- Patients requiring surgical extraction of a lower third molar.
- Patients over 18 years of age.
- Patients who have read and signed the informed consent.

#### Exclusion criteria

Systemic pathologies contraindicating dental avulsion surgery.

- Systemic pathologies contraindicating dental avulsion surgery.
- Presence of uncontrolled severe periodontitis.
- Patient’s unwillingness to take part in clinical checks.
- Smokers > 10 cigarettes per day.
- Allergy or intolerance to prescribed drugs in post-operative care.
- Patients requiring pre-operative antibiotic prophylaxis.

After enrolment, the selected patients will undergo the planned surgery, and GCF (a few minutes before surgery) and WF (at 24 h and 7 days after surgery) sampling.

They were processed and studied at the laboratories of the NEUROFARBA Department - Department of Neuroscience, Psychology, Drug Area and Child Health, Viale Pieraccini 6, 50139, Florence.

### Outcomes of the study

The concentrations of the following proteins: VEGF-A, IL-1β, IL-6, IL-10, G-CSF, TNF-α, β-NGF are expressed in pg/ml, for the following observation time: T0: just before surgery; T1: 24 h after surgery; T2: 7 days after surgery.

At T0 GCF is taken from the sulcus of the mesial or distal papilla of the element to be avulsed, to obtain a personal baseline for each patient. At T1 and T7, WF is taken from the surgical wound margin, assessing the change in protein concentration from baseline.

### Surgical procedure

A 4% articaine with adrenaline 1: 100,000 is used as anaesthetic. The incision is made and a full-thickness flap is sculpted. This is followed by osteotomy, performed with a tungsten carbide bur mounted on a turbine and abundant irrigation; odontotomy is performed with a slit drill on a turbine, when necessary. Following avulsion, the post-surgical socket is cleansed with physiological saline solution; sutures are then applied (vycril 2/0). The intraoperative evaluation form is completed in order to report relevant events that may possibly represent significant deviations from the standard protocol, thus representing an exclusion criterion. Patients will comply with the following post-operative drug therapy: amoxicillin clavulanic acid 1 g, one tablet every 12 h for 6 days, ibuprofen 600 mg, post-operatively and after 8 h. Then *as needed* not More than 3 times a day; chlorhexidine 0.20% mouthwash pure, 3 times a day and subsequent application of 1% chlorhexidine gel. The patient’s card will be filled in, in which the following will be noted: immediate, remote and pharmacological history.

### Bias

- Should the sample be contaminated with saliva or blood, it will be discarded.
- The sample will be taken in duplicate.

### Data collection

GCF is obtained at baseline from the mesial or distal gingival sulcus of the element that will undergo avulsion; WF is taken from the surgical wound margin at later observation times due to the absence of clinical wound closure [21]. Sampling is performed using a dedicated sterile paper strip: Periopaper^®^, Oraflow Inc., Hewlett, NY, USA. 2-x 13 mm [21, 22]. Tissues are kept dry by placing cotton rolls in the vestibular fornix and blowing air through the air-water syringe [23]. Each sampling is performed in the same spot in duplicate, leaving the Periopaper in place for 30 s. The positioning of the Periopaper is gentle and aims to avoid any mechanical trauma [24].

The waxed portion of the paper strip is separated from the GCF/WF-impregnated portion using sterile scissors [25]; the impregnated portion of two WF samples is transferred into a sterile test tube containing:

- sterile Dulbecco’s Phosphate Buffered Saline (PBS; 300 µl/sample; Merck, Milan, Italy) [25];
- complete Protease Inhibitor Cocktail (¼ tablet in 2.5 ml of PBS; Roche, Basel, Switzerland) [26].;
- 0.5% of bovine serum albumin (BSA; Merck, Milan, Italy).

The test tubes are then kept on ice for about an hour pending transport to the laboratory.

The samples are then subjected to elution by centrifugation (13,000 g; 10 min; 4 °C) [25]; the extracted protein supernatant is collected and stored at a temperature of −80 °C until subsequent analysis [27].

Molecular analysis is performed using a multiplex panel (Bioplex™ Cytokine Assay, Bio-Rad Laboratories, Inc.) following the manufacturer’s instructions in order to assess the concentration of each protein at each time of observation and the total quantitative level [21, 25].

### Statistical analysis

The sample size (*n* = 24) was based on feasibility considerations and is in line with similar pilot studies investigating cytokine levels in gingival crevicular fluid during wound healing. The data collected were subjected to descriptive evaluation. The concentration of each protein at each evaluation timepoint is expressed as mean ± SEM. The data were analysed using the Wilcoxon signed-rank test, which accounts for the paired nature of the measurements across timepoints. A p-value < 0.05 will be considered statistically significant. In the case of drop-outs, the *per-protocol* approach was used.

## Results

### Population

26 patients were initially enrolled in the study, but two of these did not present for follow-up. Therefore, a total of 24 patients were included and re-evaluated at all observation times. Between February 2022 and July 2023, all 24 patients (11 males and 13 females) each underwent a semi-enclosed lower third molar extraction. The mean age of the patients was 27.79 ± 7.55. The demographic data of the patients are shown in Table 1.

**Table 1:** Demographic data

Patients	n = 24
Age (average [SD])	27.96 (7.66)
Male gender (n [%])	11 (45.8)
Tobacco habit (n [%])	3 (12.5)
Allergies	0 (0)
Systemic diseases	5 (20.8)
Medications for systemic diseases	5 (20.8)

The primary reason for extraction of the lower third molar was pericoronitis, accounting for 84% of cases. In 4 out of 24 cases (16.7%), caries was observed in the lower third molar. Regarding tooth orientation, 15 elements (62.5%) were vertical, 7 (29.2%) were mesioverted, and 3 (12.5%) were horizontal. Patient 1 had acute promyelocytic leukemia and hypertension; patient 4 had myeloid leukemia and hypertension; patient 10 had epilepsy; patient 11 had atrial fibrillation; patient 12 had epilepsy. These patients required chronic drug therapy. Patient 1 was taking entecavir antivirals, cyclosporine and antihypertensives. Patient 4 was taking venetoclax and allopurinol, plus antihypertensives. Patients 10 and 12 were on antiepileptics. Patient 11 was on eliquis and canrenone. Linear regressions show no statistically significant associations between the average concentrations of molecular markers and the presence of systemic diseases or concomitant chronic therapies.

### Results

The protein distributions at the three observation times were described using box plots. Protein concentrations are expressed in pg/ml. Each box plot has a confidence interval and a mean to compare the concentrations of each protein at the three observation times.

**Fig. 1:**
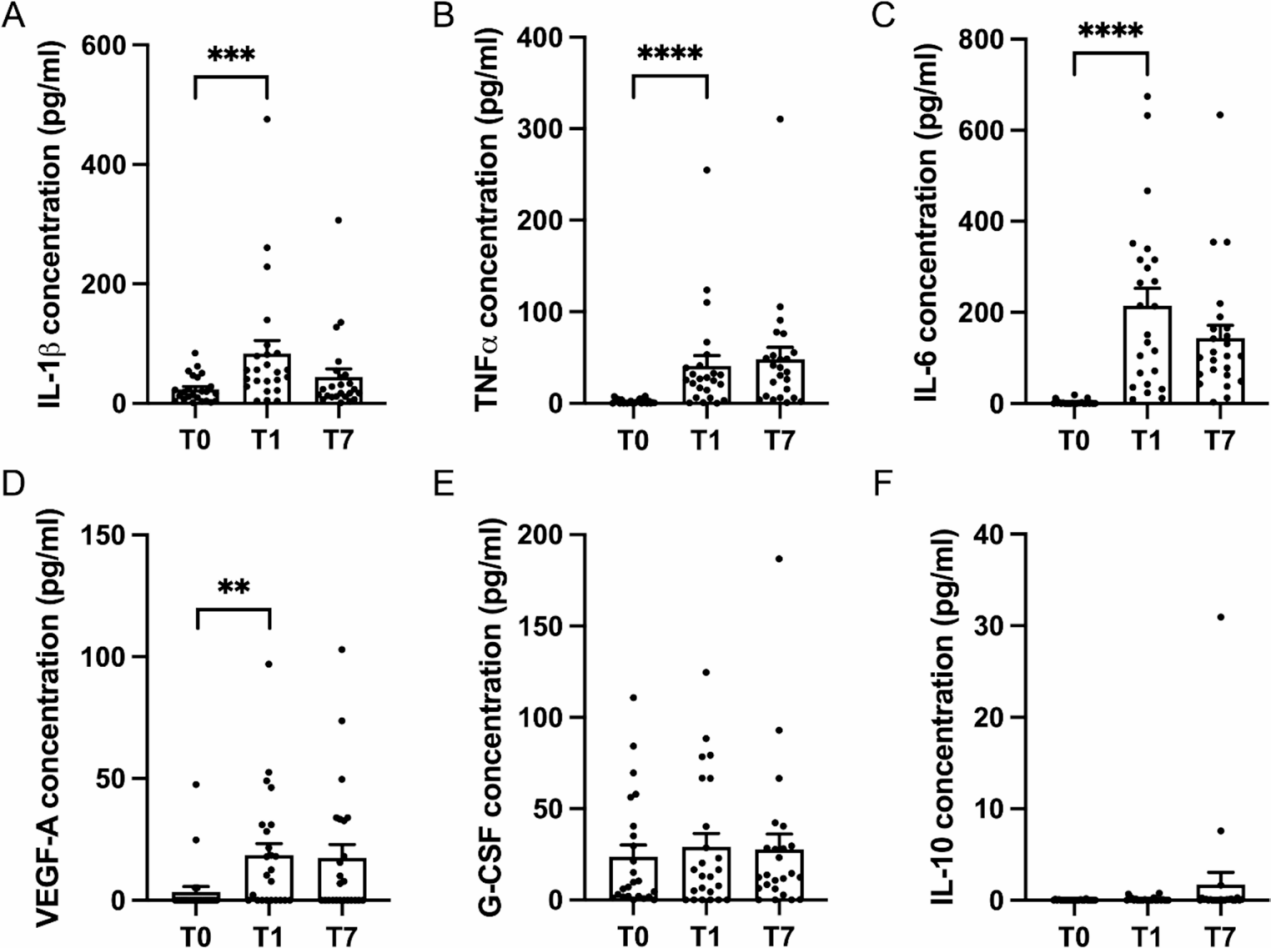
Cytokine concentrations measured in gingival crevicular fluid (GCF) at T0 and in wound fluid (WF) at T1 and T7. The graphs display the levels of (**A**) IL-1β, (**B**) TNF-α, (**C**) IL-6, (**D**) VEGF-A, (**E**) G-CSF, and (**F**) IL-10 before the surgical intervention (T0), 24 h (T1) and one week (T7) after the surgery. Data are expressed as mean ± SEM

The comparison of IL-1β concentrations between T1 and T0 reveals a statistically significant increase (*p* < 0.001), with levels rising from 23.58 ± 4.52 at T0 to 83.95 ± 21.39 at T1. This increase is followed by a decreasing trend at T7 (44.12 ± 13.46) (Fig. 1A).

Similarly, TNF-α concentrations show a statistically significant difference between T1 and T0 (*p* < 0.0001). Specifically, TNF-α levels increase from 1.82 ± 0.48 at T0 to 40.88 ± 11.27 at T1, while no significant differences are observed at T7 (48.10 ± 12.91) (Fig. 1B).

IL-6 concentrations also exhibit a statistically significant rise between T1 and T0 (*p* < 0.0001), with values increasing from 2.37 ± 0.98 at T0 to 214.76 ± 38.21 at T1. A slight, though not statistically significant, decrease is observed at T7 (143.54 ± 28.08) (Fig. 1C).

VEGF-A concentrations show a significant increase at T1 (18.50 ± 4.85, *p* < 0.01) compared to T0 (3.46 ± 2.19), while no significant difference is observed at T7 (17.45 ± 5.46) (Fig. 1D).

G-CSF levels remain relatively stable across all time points, with values of 29.13 ± 7.21 at T1 and 27.75 ± 8.27 at T2, compared to 23.79 ± 6.30 at T0 (Fig. 1E).

IL-10 concentrations are barely detectable, with no significant variations across the observation periods (Fig. 1F).

## Discussion

The aim of this study was to identify a non-invasive and reproducible method to explore the wound fluid (WF) content for biomarkers contained therein after surgical extraction of a tooth element.The proposed collection and processing technique made it possible to assess protein levels relative to baseline in each individual patient, in an efficient and non-invasive manner, confirming a known trend of molecular biomarkers in the early stages of healing. The modality of WF collection varies depending on the body district: among the most commonly used are occlusive bandaging [28], drains [17, 29], negative pressure therapy (NPWT) or the use of sterile gauze, swabs [30, 31] or adsorbent papers [32] dipped in appropriate buffer solutions. In dentistry, the use of paper-strip swabs is common for the collection of GCF or WF, especially for periodontal or peri-implant evaluation. In particular, using paper-strips, Alssum et al. [21] reported elevated levels of Ang-2, VEGF, IL-8, and TNF-α in early postoperative wounds, although their study focused on regenerative bone surgery rather than tooth extraction.

The healing mechanisms of a post-extraction socket are well known, but it is also interesting to focus on the composition of the wound fluid after oral surgery. Using the suggested method, we observed that the levels of IL-6, TNF-α and IL-1β increased statistically significantly during early healing. These trends are consistent with those previously described in the literature. The release of mediators during surgical wound healing is temporally well organised [33, 34]. Immediately after the creation of the wound, TNF-α and IL- 1 are produced at high concentrations [7], to initiate the inflammatory cascade and with chemotactic as well as stimulatory action on osteoclastic activation, B- and T-cells. IL-6 also plays a prominent role in the initiation of wound healing, so that high levels are detected in early wound healing. Consistent with the assessment of Lebeblicioglu et al. [35] it is IL-6 that shows the most significant increase in the surgical wound exudate of a Guided Bone Regeneration (GBR); Lin et al. [36] demonstrated that the lack of IL-6 expression in animals correlated with a significant delay in re-epithelialisation and granulation tissue formation. In general, the absence of these three cytokines results in delayed or impaired healing [36]. On the other hand, overexpression of these mediators results in an obstacle to regenerative procedures [9], is significantly correlated with the severity of periodontal disease [37–39] and at the same time, the pro-nociceptive action of these mediators is known [6], so that a real paradox is created regarding their role in inflammatory mechanisms [7, 13] and modulation of their concentration may be a therapeutic goal. Levels of the best-known anti-inflammatory cytokine, IL-10, are minimal and do not show significant changes at all observation times as assessed by Kaner et al. using paper-strip WF following periodontal surgery [23]. Regarding VEGF-A, its levels show a significant increase at T1, which remains elevated at T7. This increase is likely induced by IL-6, as suggested by previous research [13]. VEGF-A is recognized to have protective and pain-related properties, depending on its interaction with specific receptors, as highlighted in recent studies [40, 41] and it has been investigated as a potential biomarker for neuropathy in animal models [42]. These findings suggest that VEGF-A may also be involved in nerve-related processes during the healing of oral tissues. As a result, the sustained increase in VEGF-A aligns with its elevated levels in gingival wounds, highlighting its significant role in the repair and regeneration of both vascular and neural tissues. Other studies have shown a similar trend of VEGF, with an initial peak at the early phases of healing [21, 43, 44].

G-CSF has also been shown to be associated with an increase in keratinocyte proliferation, with improvement in skin wound re-epithelialisation [45]. In the oral cavity, G-CSF has been associated with bone resorption in periodontitis, with high levels detected in serum and gingival epithelial cells [46]. In mice, G-CSF delayed bone healing of the post-extraction socket [47], but no studies show relevant concentrations of G-CSF released within the WF of oral wounds.

β-NGF has also been researched in our study but at no time have levels been evaluated that are even slightly significant. NGF is known for its prominent role in skin wound healing; however, it is also expressed in the oral cavity, being secreted in saliva as a non-mature precursor (pro-NGF) by both salivary glands and mucosal keratinocytes: using the enzyme-linked immunosorbent assay (ELISA), human salivary NGF levels have been estimated at around 1–10 ng/mL. It is likely that pro-NGF present in saliva can only bind to its receptors on basal keratinocytes after they are exposed to the wound edges. The plasmin produced during bleeding activates pro-NGF, converting it into its mature form [13, 48].

### Limitations and future directions

To date, there are no studies performed with wound samples from extractive surgery, but they would be desirable, with a view not only to a description of the markers involved in the healing process, but also to the relationship of their concentrations with variables such as certain drugs or headmasters that could change the course and with possible clinical variables such as pain and functional limitation. The present study is not without its limitations: the sample size is small but consistent with the design of a pilot study aiming at the implementation of an observational study with a larger sample size in order to obtain results that are also clinically relevant.

## Conclusions

Under the orchestration of growth factors and cytokines the early stages of healing are crucial for successful wound healing. In this context, knowledge, control and modulation of the mediators involved can be a good therapeutic target. The present study presented an effective method for the collection and study of post-extraction wound fluid in the early stages of wound healing.

## Data Availability

The datasets generated and/or analyzed during the current study are available from the corresponding author on reasonable request.
